# Predictors of seropositivity against SARS-COV-2: a population-based seroepidemiological study

**DOI:** 10.1093/eurpub/ckac129.049

**Published:** 2022-10-25

**Authors:** L Musheghyan, S Sahakyan, Z Sargsyan, D Muradyan, L Aslanyan, N Gharibyan, A Harutyunyan, V Khachadourian, V Petrosyan

**Affiliations:** Turpanjian College of Health Sciences, American University of Armenia, Yerevan, Armenia

## Abstract

**Background:**

Data on parameters of actual exposure to SARS-CoV-2 is limited, and specific population groups might be at a higher risk of infection. In line with the growing need for large-scale investigations to determine the presence of antibodies against SARS-CoV-2 among different population groups, we conducted a nationwide assessment in Armenia.

**Methods:**

We performed a cross-sectional seroepidemiological study among the adult population in Armenia, in May-September 2021. A multi-stage cluster random sampling was performed to recruit the participants across the capital city and regions. The study had two main components: blood sampling, which took place in primary care facilities and a phone survey on socio-demographic characteristics, comorbidities, and previous history of COVID-19.

**Results:**

The number of participants included in both blood sampling and phone survey was 3483. The nationwide prevalence of SARS-CoV 2 antibodies weighted by age and gender was 66.4% with significantly higher prevalence in urban compared to rural areas (67.3% vs 59.3%, p < 0.001). Only 22.7% (n = 772) of the total sample reported a previous history of PCR confirmed COVID-19, among whom antibodies were detected in 94.2% (n = 727). In the final adjusted model, the seropositivity was associated with being female (OR = 1.60, 95% CI: 1.32; 1.92), employed (OR = 1.41, 95% CI: 1.17; 1.69), and having previous PCR confirmed COVID-19 (OR = 10.6, 95% CI: 7.39; 15.21).

**Conclusions:**

Over 66% of the population were seropositive for antibodies against SARS-CoV 2; and over ⅕ of the sample reported a previous PCR diagnosis. Factors associated with increased odds of seropositivity included gender, employment status, and place of residence. Targeted interventions are recommended to minimize the risk of infection among those groups, including vaccination and infection prevention and control measures.

**Key messages:**

• The prevalence of SARS-CoV 2 antibodies is about three times higher than the rate of infection based on PCR confirmed prevalence of COVID-19.

• Women, people living in urban areas, and those employed are at a higher risk for exposure to SARS-CoV 2.

